# Regulation of callose synthase activity *in situ *in alamethicin-permeabilized *Arabidopsis *and tobacco suspension cells

**DOI:** 10.1186/1471-2229-9-27

**Published:** 2009-03-12

**Authors:** Mari Aidemark, Carl-Johan Andersson, Allan G Rasmusson, Susanne Widell

**Affiliations:** 1 Department of Cell and Organism Biology, Lund University, Sölvegatan 35, SE-223 62 Lund, Sweden

## Abstract

**Background:**

The cell wall component callose is mainly synthesized at certain developmental stages and after wounding or pathogen attack. Callose synthases are membrane-bound enzymes that have been relatively well characterized *in vitro *using isolated membrane fractions or purified enzyme. However, little is known about their functional properties *in situ*, under conditions when the cell wall is intact. To allow *in situ *investigations of the regulation of callose synthesis, cell suspensions of *Arabidopsis thaliana *(Col-0), and tobacco (BY-2), were permeabilized with the channel-forming peptide alamethicin.

**Results:**

Nucleic acid-binding dyes and marker enzymes demonstrated alamethicin permeabilization of plasma membrane, mitochondria and plastids, also allowing callose synthase measurements. In the presence of alamethicin, Ca^2+ ^addition was required for callose synthase activity, and the activity was further stimulated by Mg^2+ ^Cells pretreated with oryzalin to destabilize the microtubules prior to alamethicin permeabilization showed significantly lower callose synthase activity as compared to non-treated cells. As judged by aniline blue staining, the callose formed was deposited both at the cell walls joining adjacent cells and at discrete punctate locations earlier described as half plasmodesmata on the outer walls. This pattern was unaffected by oryzalin pretreatment, showing a quantitative rather than a qualitative effect of polymerized tubulin on callose synthase activity. No callose was deposited unless alamethicin, Ca^2+ ^and UDP-glucose were present. Tubulin and callose synthase were furthermore part of the same plasma membrane protein complex, as judged by two-dimensional blue native SDS-PAGE.

**Conclusion:**

Alamethicin permeabilization allowed determination of callose synthase regulation and tubulin interaction in the natural crowded cellular environment and under conditions where contacts between the cell wall, the plasma membrane and cytoskeletal macromolecules remained. The results also suggest that alamethicin permeabilization induces a defense response mimicking the natural physical separation of cells (for example when intercellulars are formed), during which plasmodesmata are transiently left open.

## Background

The cell wall polymer callose (1,3-β-D-glucan) is normally synthesized at specific developmental events, like in the cell plate [1,2] and in pollen tube walls [3]. Callose is also deposited at plasmodesmata [4,5] and at sieve plates [6] to limit intercellular transport, often as a response to developmental cues or environmental signals, e.g., wounding and pathogen attack [7-9]. Callose deposition reinforces the cell wall at the site of the attack [10,11], but callose can also be found at plasmodesmata in neighboring non-infected cells to limit spread of a fungal infection in resistant cultivars [12]. Exposure to aluminum also induces callose production [13,14] sometimes to occlude plasmodesmata [15,16].

Genes encoding callose synthases (*GSL*) [17-19] have now been identified in several plant species. In *A. thaliana *as much as 12 callose synthase genes have been identified [18]. Biochemical studies have indicated that at least some *GSL *genes can produce proteins capable of synthesizing callose [20].

Callose synthases use UDP-glucose as glucose donor to the growing polymer chain [21] similar to cellulose synthases (which form 1,4-β-D-glucan) although callose production appears to dominate in most *in vitro *experiments [22,23]. It was earlier believed that the two polymers were produced by one enzyme, which switched to callose synthesis *in vivo *upon wounding or during extraction to allow enzyme activity determinations [5,23]. The binding site for UDP-glucose for callose synthase (as well as cellulose synthase) is on the cytoplasmic side of the plasma membrane, and is thus inaccessible to direct assays in intact cells. To overcome this permeability barrier, detergents have been added to cells or isolated plant plasma membranes. This may, however, also create problems since the functional units are membrane-bound protein complexes [24-26] which could be sensitive to changes in their membrane environment like partial delipidation of the enzymes and separation of complexes. For example, the detergent Triton X-100 severely inhibited callose synthase activity in plasma membranes from oat root and cauliflower inflorescences [27].

Despite such problems, callose as well as cellulose synthesis have successfully been monitored with isolated proteins after solubilization of microsomal membranes with detergents *e.g*., digitonin, Brij 58, CHAPS or taurocholate [24,28-32]. The use of sucrose rather than UDP-glucose as substrate, led to less callose and more cellulose formation. Here, sucrose was probably metabolized by sucrose synthase to yield UDP-glucose [29]. The assay conditions for the two activities differ, *e.g*., Mg^2+ ^ions favor cellulose synthesis, whereas callose synthesis depended on the presence of Ca^2+ ^[29,33,34].

In the cell, microtubules control the deposition of cellulose by guiding the movement of the cellulose synthases in the plasma membrane [35,36]. In contrast to cellulose, callose is usually relatively amorphous. However, using plasma membrane sheets from tobacco BY-2 protoplasts isolated in the presence of taxol to stabilize microtubules, the callose was deposited in oriented microfibrils [37]. If the preparation was done in the presence of propyzamide (disrupts microtubules) instead of taxol, the product was deposited in diffusely distributed masses, suggesting that microtubules are needed to orient callose deposition at least with protoplasts [37]. There are also indications that microtubules affect callose production in the cell plate, at least indirectly. DRP1A, a phragmoplastin-like protein, was observed to associate with Golgi-derived vesicles transported along microtubules to the growing cell plate [38], and phragmoplastins interact with UDP glycosyl transferase, which probably is part of the cell plate callose synthase complex [18,26]. Deposition of callose in the cell plate was reported to be tightly linked to the depolymerization of microtubules [39].

Microtubules are sensitive to changes in the cellular environment as part of their dynamic function. Therefore, the *in vitro *conditions previously used to study callose synthesis probably deviate from *in vivo *conditions with respect to cytoskeleton associations. The microtubule-plasma membrane-cell wall continuum is broken when the plasma membrane is solubilized. Therefore, alternative ways to investigate callose synthesis, where the interior of the cell is minimally disrupted and the cell wall is still present, are highly needed as complements to detergent solubilization. One possibility is to use the channel-forming molecule alamethicin. This is a 20 amino acid amphiphilic polypeptide from the fungus *Trichoderma viride *[40], which can be used to permeabilize biological membranes [41]. It inserts into membranes when applied to the positively charged side, and forms low-specificity ion channels with10 Å pore size [42,43]. These pores allow the passage of small charged molecules like ATP and NADH while being impermeable to macromolecules like folded proteins [44,45]. This stands in contrast to the holes formed by digitonin through which proteins can pass [46]. Alamethicin is gentle regarding side effects on membrane enzyme systems (e.g. since the mitochondrial electron transport chain can be assayed, protein complexes are not separated or delipidized and lipophilic ubiquinone is not extracted [44]), whereas a detergent like digitonin will bind hydrophobic surfaces and molecules in membranes that it can permeabilize. In tobacco Bright Yellow 2 (BY-2) suspension cells, alamethicin permeabilized the plasma membrane and the inner mitochondrial membrane but not the tonoplast, allowing direct activity measurement of glycolytic and mitochondrial enzymes. Consistently, cells treated with alamethicin were depleted in metabolites within 10 min, leading to a sharp decrease in respiration. When removing alamethicin from treated cells, a large subset of cells were still viable and regained the ability to divide [47].

Here we have explored the potential use of alamethicin for permeabilization of *A. thaliana *Columbia (Col-0) and tobacco BY-2 cells to measure synthesis of cell wall polymers. In the presence of an intact cell wall, alamethicin permeabilized Col-0 plasma membrane, the inner mitochondrial membrane and the plastid envelope in virtually all cells in the treated population. This *in situ *system allowed measurement of callose synthesis, and thus describing its spatial distribution in the cells and the regulation of callose synthesis by the polymerization state of tubulin. This connection was strengthened by the observation that tubulin and callose synthase co-migrated as a protein complex during two dimensional blue native SDS-PAGE.

## Results

### Alamethicin permeabilization of Col-0 and BY-2 cells

**Figure 1:**
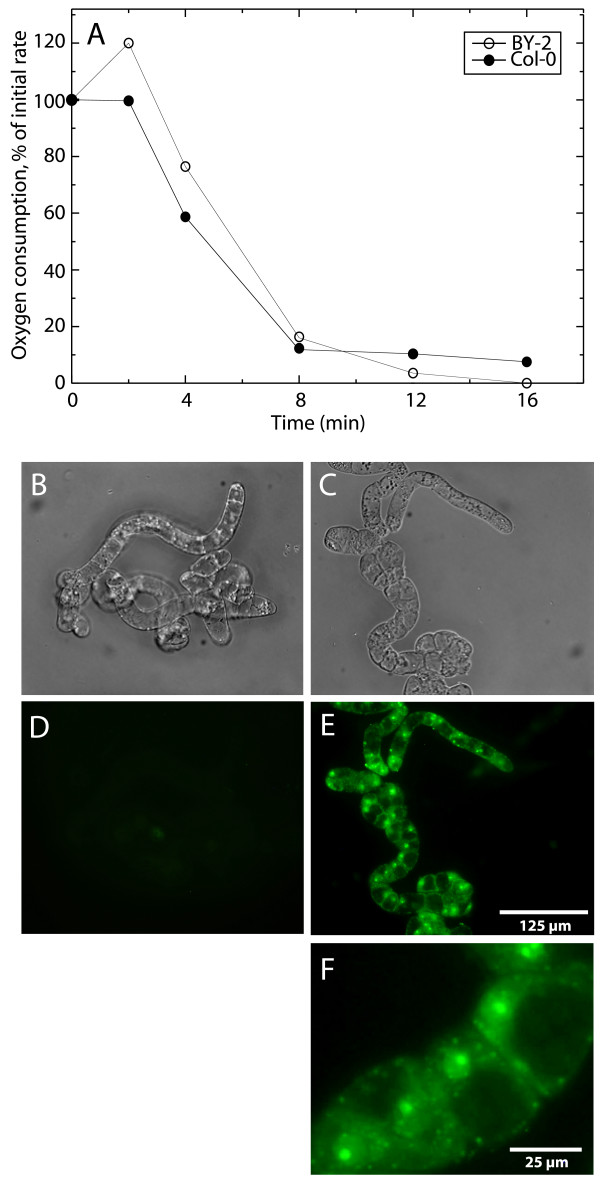
**Alamethicin permeabilization of Col-0 cells**. (A) Oxygen consumption in Col-0 and BY-2 cells after addition of 20 μg ml^-1 ^alamethicin. Points represent the rate of oxygen consumption relative to the control rate prior to alamethicin addition. (B-F) Visualization of alamethicin permeabilization of Col-0 cells by Yo-Pro staining. Bright field microscopy images of untreated (B) and alamethicin-permeabilized (C) cells as well as fluorescent images showing Yo-Pro staining of untreated (D) and alamethicin-permeabilized (E-F) cells. (F) shows a close up of (E).

It was previously shown that alamethicin could be used to permeabilize BY-2 cells [47]. To enable the use of *A. thaliana *cells in addition to BY-2 and to investigate the regulation of callose synthesis, we wished to establish if Col-0 suspension cultured cells were similarly permeabilized by alamethicin. A decrease in respiration (oxygen consumption) by metabolite depletion was found also with Col-0, and the time required to abolish respiration was around 10 min for both BY-2 and Col-0 cells (Fig. 1A). Treatment of Col-0 cells with alamethicin for 10 min also allowed the membrane-impermeable nucleic acid stain Yo-Pro to mark nuclei and organelles with uniform staining of the whole cell population (Fig. 1B–F). A virtually identical staining was produced by the membrane-impermeable nucleic acid stain propidium iodide, as observed by perfectly overlapping double staining (results not shown).

**Figure 2:**
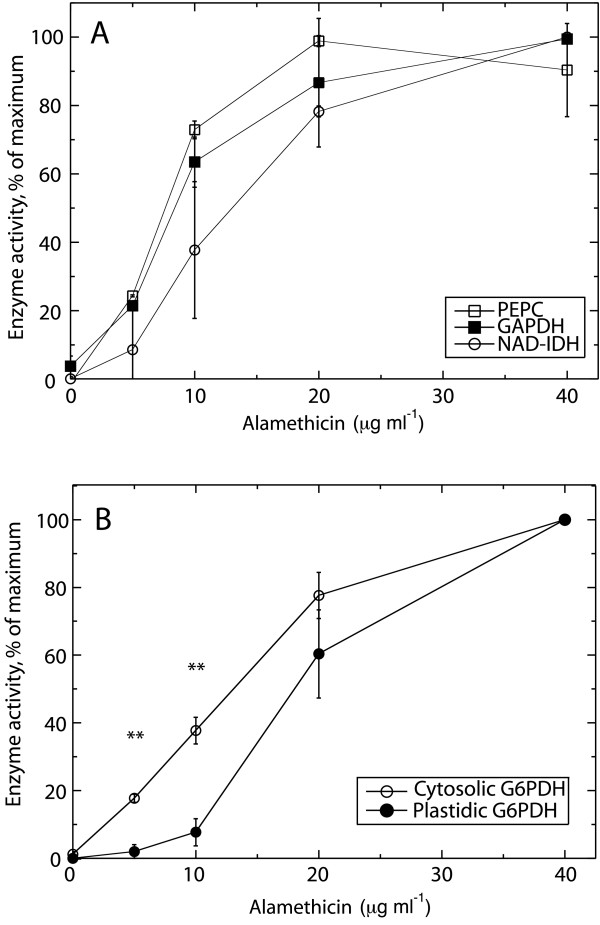
**Activities of metabolic enzymes in alamethicin-permeabilized Col-0 cells**. Rates are expressed as percent of the highest rate in each experiment. (A) Effect of alamethicin on activities of PEPC, GAPDH, and NAD-IDH. The average maximum activity was for PEPC 480 ± 220 nmol min^-1 ^g^-1^(FW), for GAPDH 1650 ± 300 nmol min^-1 ^g^-1^(FW), and for NAD-IDH 210 ± 140 nmol min^-1 ^g^-1^(FW). Averages of two independent experiments with error bars representing S.D. are shown. (B) Effect of alamethicin on activities of cytosolic and plastidic G6PDH. Averages are shown for three independent experiments with error bars representing S.E. The average maximum activity was 260 ± 50 and 420 ± 160 nmol min^-1 ^g^-1^(FW) for cytosolic and plastidic G6PDH, respectively.

The apparent activities of NAD-glyceraldehyde-3-phosphate dehydrogenase (GAPDH; marker for cytosol), phosphoenolpyruvate carboxylase (PEPC; marker for cytosol), and NAD-isocitrate dehydrogenase (NAD-IDH; marker for mitochondria), increased in Col-0 cells treated with increasing concentrations of alamethicin, indicating permeabilization of the plasma membrane and the inner mitochondrial membrane (Fig. 2A). The maximum activity was approached using between 20 and 40 μg ml^-1 ^alamethicin, and more than 60% of maximum activity was reached already using 10 μg ml^-1 ^of alamethicin for cytosolic enzymes. The activities of GAPDH, PEPC and NAD-IDH in alamethicin permeabilized cells were 90–100% of the activities measured after solubilizing with 0.1% (v/v) Triton X-100 (results not shown).

The activities of cytosolic and plastidic glucose-6-phosphate dehydrogenase (G6PDH) also increased with increased alamethicin, but not identically. Cytosolic G6PDH activity was detected at lower alamethicin concentrations compared to that of the plastidic form (Fig. 2B). This difference was significant when 5 or 10 μg ml^-1 ^of alamethicin was used (Fig. 2B). The maximum activity measured for the plastidic G6PDH was higher than that of the cytosolic G6PDH in alamethicin-permeabilized cells (Fig. 2B, legend). Triton X-100 (0.1%) severely inhibited the plastidic enzyme, resulting in activities being 20 ± 10% of those obtained after alamethicin permeabilization. In contrast, no inhibitory effect by Triton X-100 was found for the cytosolic enzyme. The results thus show that alamethicin homogenously permeabilizes a population of Col-0 cells with respect to plasma membrane, mitochondria and plastids.

### Characterization of callose synthesis in alamethicin-permeabilized cells

**Figure 3:**
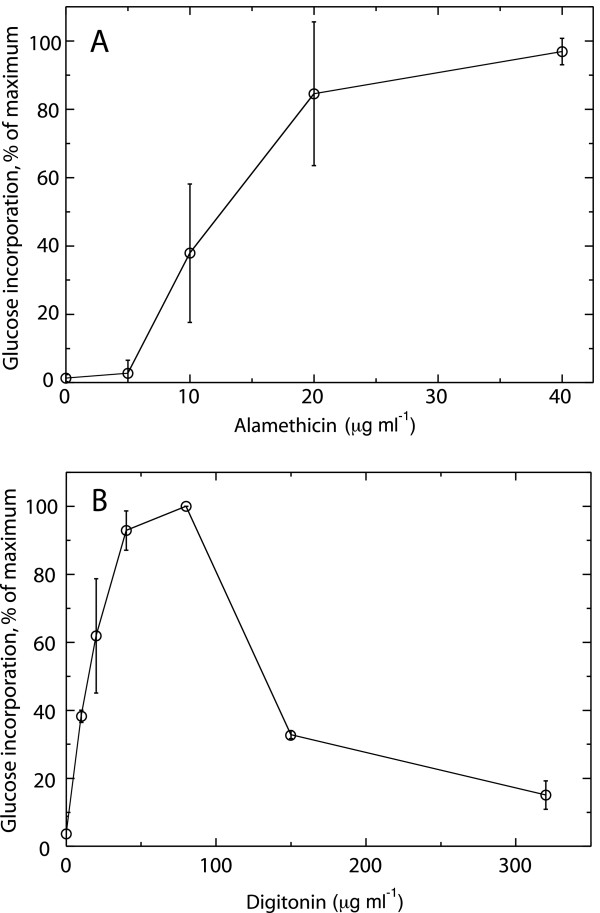
**Callose synthesis measured in cells permeabilized with alamethicin or digitonin**. Values are normalized to maximum activity in each experiment and error bars represent S.D. (A) Effect of increasing concentrations of alamethicin on callose synthesis. The average of maximum activity was 53 nmol min^-1 ^g^-1^(FW) and values represent the mean of two to four independent experiments. (B) Callose synthesis in the presence of digitonin. Data points for digitonin are averages of two independent experiments and the average of maximum activities was 16 nmol min^-1 ^g^-1^(FW).

Having seen that Col-0 cells were efficiently permeabilized by alamethicin in a manner similar to what was previously reported [47], we next wanted to investigate whether this system could be used to monitor the plasma membrane-bound enzyme callose synthase *in situ*. Digitonin was chosen for comparison when following UDP-glucose incorporation, since this agent has been used in many investigations. The activity measured (incorporation of labeled glucose from UDP-glucose into ethanol- and ammonium acetate insoluble products) using alamethicin (present 10 min before assay and during the 10 min assay) was generally of similar magnitude or higher than that measured using digitonin. The shape of the alamethicin curve was sigmoid for UDP-glucose incorporation (Fig. 3A) as for the metabolic enzymes (Fig. 2), suggesting a cooperativity between the alamethicin molecules during channel formation. In contrast, the digitonin curve was hyperbolic in the lower concentration range, while at higher digitonin concentrations the activity was severely inhibited (Fig. 3B).

**Figure 4:**
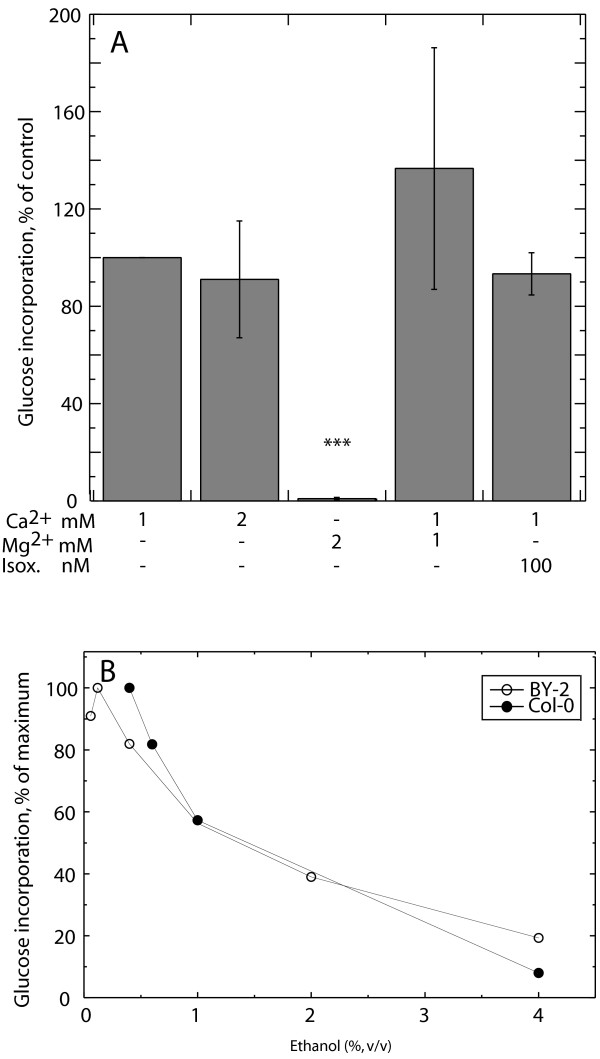
**Characterization of callose synthase activity**. (A) Experiments were performed on Col-0 cells treated with 20 μg ml^-1 ^of alamethicin in assay medium complemented with various amounts of Ca^2+^, Mg^2+ ^and isoxaben (Isox.). Activities for each independent experiment are presented relative to the activity in the presence of 1 mM Ca^2+^. The average activity with 1 mM Ca^2+ ^was 45 nmol min^-1 ^g^-1^(FW). Values represent averages of at least three independent experiments except for the 2 mM Mg^2+ ^experiment which was determined twice. (B) The effect of ethanol addition of callose synthase activity in Col-0 and BY-2 cells. Each curve represents one independent experiment. The maximum activity was 45 nmol min^-1 ^g^-1^(FW) in Col-0 and 10 nmol min^-1 ^g^-1^(FW) in BY-2 cells.

To further characterize UDP-glucose incorporation in alamethicin-permeabilized Col-0 cells we varied the concentrations of Ca^2+ ^and Mg^2+ ^in the assay. The activity was strongly stimulated by Ca^2+^. Substituting the Ca^2+ ^with Mg^2+ ^abolished the activity. The highest activity was obtained after addition of both 1 mM Ca^2+ ^and 1 mM Mg^2+ ^(Fig. 4A). No effect was obtained when the cells were preincubated with the cellulose synthase inhibitor isoxaben (Fig. 4A). The lack of inhibition by isoxaben together with the stimulation by Ca^2+ ^addition indicate that callose synthase activity indeed was measured [31,48,49].

It was observed that ethanol negatively affected the measured callose synthase activity. Some ethanol (0.06% or 0.12% [v/v]) was always present in the assay as solvent for alamethicin). With increasing concentration, ethanol substantially decreased the activity in Col-0 and BY-2 cells (Fig. 4B). Ethanol inhibition of callose synthesis was also observed in digitonin-permeabilized Col-0 cells (results not shown), showing that the inhibition was not due to effects on alamethicin channel formation.

### Callose synthase and microtubules in alamethicin permeabilized cells

**Figure 5:**
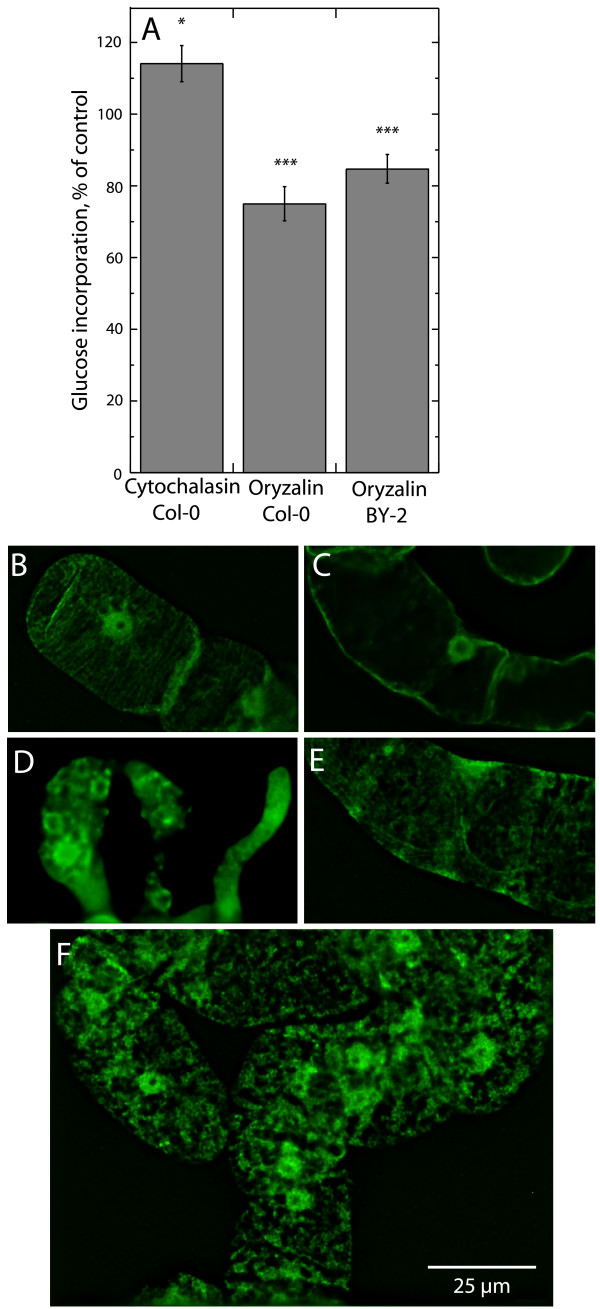
**Effect of cytoskeleton modifying agents on callose synthase and cytoskeleton structure**. (A) Callose synthase activity after treatment of 3–5 day old cells with cytoskeleton-affecting agents. Activities are given as per cent of the DMSO control. The average of the activity for the DMSO assays was 19 nmol min^-1 ^g^-1^(FW) for Col-0 and 27 nmol min^-1 ^g^-1^(FW) for BY-2 cells. The values are means of three or more independent experiments. Error bars represent S.E. (B-F) Organization of the microtubules after different treatments. Deconvoluted fluorescence images are shown for cell cultures that were untreated (B), pretreated with 1 μM oryzalin (C) for 2 h, 0.1% (v/v) Triton X-100 (D) for 30 min, 0.016% (v/v) digitonin (E) for 30 min or with 20 μg ml^-1 ^alamethicin (F) for 10 min. The untreated, detergent-treated and alamethicin-treated samples were washed and diluted in Assay medium 2 prior to fixation, while oryzalin treated samples were fixed directly in growth medium. DMSO-containing controls for the oryzalin treatment showed a highly similar pattern to the untreated control (B).

To investigate the role of the cytoskeleton on callose synthesis, cells were preincubated with 1 μM oryzalin for 2 h to inhibit microtubule polymerization prior to alamethicin permeabilization and assay. Treated cells (oryzalin being present during pretreatment, permeabilization and assay) showed significantly lower callose synthase activity compared to control (DMSO-treated) cells (Fig. 5A). Omitting oryzalin during permeabilization and assay gave similar inhibition (81 ± 7% of DMSO control) showing that oryzalin did not interfere with the assay. In contrast to the oryzalin effect, pretreatment with 10 μM cytochalasin, which inhibits actin polymerization, lead to somewhat increased activity (Fig. 5A). A slightly but not significantly lower value (92 ± 5%) was seen after incubation with 5 μM taxol, known to stabilize microtubules. The lowered activity measured after preincubation with oryzalin suggested that the presence of polymerized tubulin was important for maximum callose synthesis in both Col-0 and BY-2 cells.

Immunofluorescence studies of control (DMSO-treated) Col-0 cells, using β-tubulin antibodies, showed the presence of parallel microtubules around the cell periphery (Fig. 5B). In oryzalin-treated cells, microtubules were no longer present and β-tubulin was distributed in the cytosol, probably as unpolymerized subunits (Fig. 5C). Not surprisingly, a cellular collapse was observed after Triton X-100 addition to living cells (Fig 5D). The cells also appeared damaged after digitonin treatment. The microtubule organization in parallel strands seen in the control (Fig. 5B) was lost with digitonin (Fig. 5E). At the same time, the pattern with digitonin was strongly deviant from the distribution of depolymerized tubulin seen after oryzalin treatment (Fig. 5C). The polymeric tubulin remaining after digitonin treatment lacked orientation, probably reflecting a partial depolymerization taking place (Fig. 5E). Similarly, after addition of alamethicin (Fig 5F), polymeric tubulin was seen reorganized into thicker and more netlike structures, which were somewhat punctate. Inclusion of Mg^2+ ^during alamethicin permeabilization resulted in a similar pattern (results not shown). Tubulin polymerization by itself was not affected by the presence of the peptide, as seen by light scattering with purified tubulin (results not shown).

**Figure 6:**
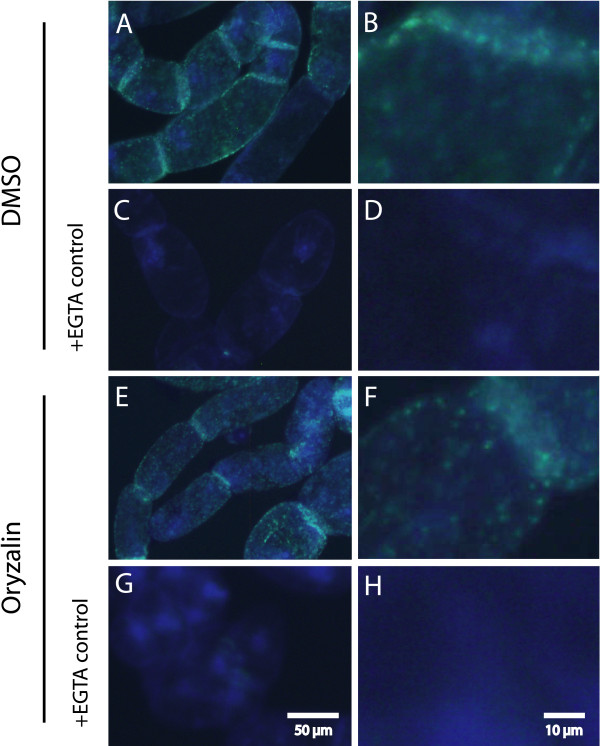
**Aniline blue staining of alamethicin-treated BY-2 cells**. (A, C) shows cells pretreated with DMSO as solvent control while in (E, G) cells have been pre-treated with oryzalin. A callose synthase reaction was performed before staining, but in C and G, EGTA was added before the start of the reaction, to chelate the Ca^2+ ^present. (B), (D), (F) and (H) are close ups for (A), (C), (E) and (G) respectively. Bars in (G) and (H) are size markers for the respective columns.

To find out if the pretreatment with oryzalin also affected callose synthesis qualitatively, alamethicin-permeabilized BY-2 cells were stained with aniline blue. Callose was deposited in spots, sometimes in rows, on outer walls (walls facing the medium) as well as in larger quantity at cell-cell connections (Fig. 6A). Hardly any callose was produced if EGTA was present in the assay to chelate Ca^2+ ^(Fig. 6C). Unpermeabilized cells showed no staining BY-2 cells pretreated with oryzalin showed a similar dual distribution of callose deposition. Due to the heterogeneity of the cell population with regard to callose deposition it was not possible to quantify callose production. However, visual inspection indicated a generally lower staining in oryzalin treated cells (Fig. 6A, E). A similar pattern of callose deposition was also observed in permeabilized Col-0 cells (results not shown).

### Native gel electrophoresis of isolated plasma membranes

**Figure 7:**
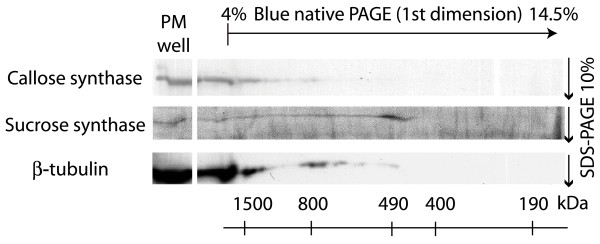
**Two-dimensional blue native/SDS-PAGE and immunoblotting of solubilized BY-2 plasma membranes**. Native, n-octyl-β-D-glucoside-soluble, plasma membrane protein complexes were separated in a first dimension using Blue native PAGE. After denaturation, the complexes were thereafter separated into their subunits in a second dimension using SDS-PAGE. After separation, callose synthase (180 kDa), sucrose synthase (90 kDa) and β-tubulin (50 kDa) were detected by immunoblotting in separate blots with the respectively specific antibodies. The figure is a composite of these separate blots. Native molecular masses for the first dimension are denoted in kDa below the blots. The upper line depicts the start and direction of the first dimension separation gel.

The data presented above indicate an interaction between callose synthase and microtubules/tubulin that remained after alamethicin permeabilization. To further test this possible interaction, we used blue native SDS-PAGE to separate plasma membrane protein complexes isolated from untreated BY-2 cells, as was successfully done earlier with spinach leaf plasma membranes [25]. In BY-2 cells, callose synthase appeared in two different protein complexes with masses of approximately 1500 kDa and at 800 kDa, each comigrating with tubulin, that was more abundant at the same masses (Fig. 7). The comigration suggests that callose synthase and tubulin are part of the same complexes through a relatively strong physical interaction, sufficient for the binding to remain during isolation and gel analysis. A mass of around 800 kDa for the callose synthase complex was also found with spinach leaf plasma membranes [25]. Sucrose synthase, on the other hand, was not here associated with callose synthase but found in a separate complex, with a molecular mass between 400 and 500 kDa (Fig. 7), consistent with the enzyme being a tetramer *in vivo *[50].

## Discussion

Much information on the synthesis of callose has been obtained in relatively dilute *in vitro *assays using isolated enzymes or membrane fractions. However, in the cell most processes are characterized by tightly controlled, more or less transient, protein interactions that take place in a crowded and compartmentalized environment. There has therefore been a need for good protocols for *in situ *investigations to further approach cellular conditions. In earlier experiments we used alamethicin permeabilization of tobacco BY-2 cells to measure activities of enzymes of the primary metabolism in the cytosol and in mitochondria [47]. We here show that alamethicin efficiently and homogeneously permeabilizes *A. thaliana *Col-0 cell populations, and that also plastids are permeabilized, albeit at somewhat higher concentrations than needed for the plasma membrane. The permeabilization by alamethicin of the inner envelope membrane shown here, agrees with what can be predicted from membrane potential orientations [43]. Similarly, predictions that the tonoplast should be permeabilization-resistant have been experimentally verified [47]. We have used this system for studies on the regulation of plasma membrane-bound callose synthesis.

Callose in the cell wall is synthesized by plasma membrane-bound multiprotein complexes and products are most often deposited in a preexisting wall. Thus, to learn about how these processes are regulated, it is desirable to have the plasma membrane-cell wall continuum intact. We here show that callose synthase activity could be determined in Col-0 and BY-2 cells permeabilized with alamethicin as well as with digitonin but that the activities using alamethicin were higher. Digitonin inhibited callose synthesis especially at higher concentrations, and maximum activation was probably never reached (i.e. enzyme capacity was not determined). The inactivation was likely due to digitonin producing large holes [51] that should deteriorate membranes, and possibly by binding hydrophobic surfaces of proteins. The digitonin concentrations used in earlier studies ranged from 0.01% [29] to 1% [28,48], i.e. in the range where the callose synthase in our study changes from being activated to severely inhibited (Fig. 3B). In contrast, alamethicin concentrations up to 60 μg ml^-1 ^did not inhibit callose synthase activity (Fig 3A). The small size of the alamethicin pore (10 Å) compared to the less defined large holes produced by digitonin (80–100 Å), will also allow a better maintenance of compartment separation, since folded proteins can pass through membranes after permeabilization with digitonin, but not alamethicin [44,51]. For example, 8 μM digitonin (10 μg ml^-1^) was enough to deplete rat hepatocytes of cytosolic lactate dehydrogenase [46].

We noted a sharp decrease of callose synthase activity upon addition of ethanol. Ethanol is synthesized naturally during anoxia [52] and one might expect that an increased need for glycolytic breakdown of sucrose to satisfy cell energy demands would decrease the shuttling of UDP-glucose towards cell wall synthesis. During anoxia, 9 to 40 μmol g^-1 ^(FW) ethanol have been observed [53,54], though being highly volatile, ethanol determinations in tissues should be expected to be underestimations [55]. The 1% ethanol concentration needed to achieve strong callose synthase inhibition (around 50%) corresponds to 140 μmol g^-1 ^(FW). Therefore, some inhibition could likely be present even at physiological concentrations of ethanol, especially if ethanol diffusion out of anoxic cells would be partially limited. Furthermore, the callose synthase assay employed here could not be performed in the complete absence of ethanol since it was used as solvent for alamethicin (final concentration of ethanol in most experiments was 0.06% [v/v]). Therefore, the potential inhibitory effect of low concentrations of ethanol may have been underestimated.

Aniline blue staining indicated that callose was deposited in spots over the cell surface, especially in walls connecting cells, but also in outer walls (walls facing the assay medium). Staining was found only after alamethicin permeabilization and addition of Ca^2+ ^and UDP-glucose (Fig. 6), consistent with the conditions in the *in vitro *incorporation assay and the requirements for callose synthesis in isolated BY-2 phragmoplasts [39]. The spot-like callose deposits in outer walls resemble structures seen earlier in aluminium-exposed cell suspensions of tobacco [56] as well as in *A. thaliana *cell suspensions [57]. Based on the colocalisation of callose and the ER protein calreticulin in isolated cell walls, the spots were suggested to be half-plasmodesmata [57] which, however, must be nonfunctional with respect to transport. In regenerating *Solanum nigrum *protoplasts, discontinuous half-plasmodesmata were initially formed on the outer walls at regions of ER-entrapment, which disappeared as the wall was reformed, unless they were fused with half-plasmodesmata of other cells [58]. In filamentous cell suspensions of *A. thaliana*, a wound-like response was induced by arabinogalactan-binding Yariv phenylglucosides, including the formation of plug-like callose deposition on outer walls [59]. However, due to low magnification, the possible presence of also punctate callose staining at outer walls cannot be excluded. We found that generally less callose was deposited both at cell-cell and outer walls after incubation with oryzalin (Fig. 6), *i.e*., the lowered activity was not an indirect consequence from effects of microtubule disruption on mitosis and cytokinesis.

Using immunofluorescence detection of tubulin, we could observe that the microtubules had become reorganized after alamethicin permeabilization, but detected tubulin was still polymeric. This suggests that the microtubules were partially, but far from fully depolymerized. Callose synthase activity was lower in cells preincubated with oryzalin prior to assay (Fig. 5A) for both Col-0 and BY-2 cells. The tubulin reorganization induced by permeabilization, and associated Ca^2+ ^influx, may thus reflect a regulatory interaction between callose synthase and a tubulin network in the process of being restructured. Taken together the results suggest that the native plasma membrane protein complexes containing callose synthase and tubulin seen using blue native SDS-PAGE (Fig. 7), reflected functional units *in situ*. Furthermore, their interaction must be relatively strong since it remained during native gel electrophoresis (Fig. 7). In contrast, sucrose synthase which has been hypothesized also to interact with callose synthase [17] to deliver substrate for the enzymatic reaction, was not found to be associated with the complex. This strong interaction between callose synthase and tubulin is in line with that a pool of plasma membrane-bound tubulin showed hydrophobic properties suggesting a tight interaction with the membrane [60]. The improved maintenance of the cytoskeleton-enzyme continuum allowed by alamethicin (as compared to detergents) may be useful also for investigating cytosolic carbohydrate metabolism enzymes, whose activity is affected by presence of cytoskeletal proteins [61-64]

In the work presented here, the effect of oryzalin on callose synthesis was quantitative rather than qualitative. This is opposite to findings reported earlier on the synthesis of glucan (i.e. callose) microfibrils using membrane sheets isolated from BY-2 protoplasts [37]. In those experiments, the total production of glucan polymers was independent on the presence of microtubules. However, microtubules were needed to control the orientation of the glucan microfibrils formed, i.e., ordered fibrils were obtained if the microtubules were stabilized with taxol but not when these were destabilized by propyzamide. The contrasting results probably reflect the different situations in a cell (this investigation) compared to a protoplast [37] during the deposition of cell wall material.

The punctate distribution of the polymeric tubulin seen in alamethicin-permeabilized cells (Fig. 5C) resembles that of the callose deposits seen using aniline blue staining (Fig. 6). It is therefore possible that the callose deposits coincide with the areas where the original microtubules were in contact with the plasma membrane. That such contacts involve plasmodesmata have been indicated in several previous reports. In *N. benthamiana *leaves infected with tobacco mosaic virus, the movement protein colocalized with ER and was targeted to punctate sites related to plasmodesmata in a microtubule-dependent manner [65]. Also, the microtubule-bundling protein AtMAP65-5 colocalized with plasmodesmata in newly formed cell walls, suggesting that it is an integral part of the plasmodesmal complex [66]. Other cytoskeletal elements (*e.g*., other microtubule-associated proteins, actin and myosin) may also be part of the machinery regulating intercellular trafficking [67,68].

It is intriguing that a general permeabilization by a peptide agent induces a spatially distinct response, *i.e*. callose synthesis located at specific points. After mechanical isolation of bundle sheath cells of C_4 _grasses, non-selective channels were formed with an exclusion limit of ca 1 kDa, consistent with open half-plasmodesmata [69,70]. In the plant, separation of cells occurs as a natural stage of development, especially in tissues with large intercellulars, and transiently open half-plasmodesmata are inevitably formed. Our results therefore indicate that the alamethicin-induced permeabilization mimics the signal for the induction of a defense response against plasmodesmal leakage. The response eventually leads to the closing of plasmodesmata, assisted by callose formation being induced by the elevated Ca^2+^. This plasmodesmal closing could be important for cell survival after physical separation of previously connected cells but also as a response to other lethal challenges to neighboring cells. We have previously observed that BY-2 cells can be recultivated after alamethicin permeabilization, *i.e*., plant cells can survive a substantial permeabilization [47]. It must likewise be assumed that cells in a tissue can survive the temporary permeabilization consequential to the formation of half-plasmodesmata upon separation of cells. Taken together, our results opens up new perspectives regarding how plant cells respond to the temporary permeabilizations that are inevitable during development, e.g., during the schizogenic formation of intercellular spaces.

## Conclusion

The channel-forming peptide alamethicin permeabilized plasma membrane, mitochondria and plastids in cultured cells of Arabidopsis and tobacco. This allowed *in situ *activity analysis of callose synthase, a complex plasma membrane-located enzyme, under conditions where the continuous interactions cell wall -plasma membrane -cytoskeletal macromolecules remained. In contrast, callose synthase in these cells was severely inhibited by digitonin, another often used permeabilization agent. Blue native gel electrophoresis of isolated plasma membranes indicated that callose synthase and tubulin were part of the same protein complex. Callose synthase activity was consistently inhibited in cells pretreated by oryzalin to destabilize the microtubules. However, irrespective of oryzalin pretreatment, callose was deposited in a punctate manner at walls between cells and at outer walls. The pattern of this deposition resembled half-plasmodesmata. The results thus suggest that alamethicin permeabilization induces a defense response to a transient permeabilization taking place during the natural physical separation of cells.

## Methods

### Plant material

Cells of *Arabidopsis thaliana *Col-0 were cultured in 50 ml of Murashige and Skoog basal salts (Duchefa, Haarlem, the Netherlands) medium supplemented with 3% sucrose, Gamborg's B5 vitamins, 3 mM MES and 1 mg l^-1 ^2,4-dichlorophenoxyacetic acid (pH 5.7). *Nicotiana tabacum *BY-2 cells were grown as previously described [47]. The cultures were grown at 24°C in constant darkness at 125 rpm on a rotary shaker and subcultured every seventh day. The cells were harvested for experiments and isolation of membrane fraction during their exponential growth phase (350 – 450 mg fresh weight cells per ml medium) unless otherwise stated. In some experiments, cells were pretreated with either 10 μM cytochalasin D (Sigma, St. Louis, MO, USA), 1 μM oryzalin (Dow Elanco, Indianapolis, IN, USA), 5 μM taxol (Sigma) or the corresponding volume of the solvent DMSO (maximum 0.2% v/v), added to the growth medium two hours before the start of the experiment.

### Oxygen electrode measurements

For oxygen consumption measurements cells were diluted in Assay medium 1 (100 mM HEPES/KOH, 100 mM mannitol, 50 mM KCl, 4 mM MgCl_2 _and 1 mM EGTA, pH 7.5) to 40 mg (FW) ml^-1^. A 1 ml Clark Oxygen Electrode (Rank Brothers, Cambridge, U.K.) was used to measure respiration. To inhibit peroxidase-mediated cell wall NAD(P)H oxidation, 192 U/ml catalase (Sigma) was present in the medium during the measurements [47].

### Yo-Pro and propidium iodide staining of Col-0 cells

Col-0 cells were diluted to 40 mg (FW) ml^-1 ^in Assay medium 1. Cells were permeabilized by incubation in 20 μg ml^-1 ^of alamethicin (Sigma) for 10 min at room temperature before staining. Staining with Yo-Pro-1 (Molecular Probes Inc, Carlsbad, CA, USA) and propidium iodide (Molecular Probes Inc.) was conducted at the manufacturer's recommended concentrations, 0.1 and 1.5 μM, respectively, during the last 5 min of alamethicin permeabilization.

Fluorescence microscopy was performed using a GFP-filter (excitation at 450–490 nm, emission at 500–550 nm) for the Yo-Pro-1 stain and a G-2A-filter (excitation at 510–560 nm, emission above 590 nm) for the propidium iodide stain in a Nikon-Optiphot-2 microscope (Nikon Corporation, Tokyo, Japan). A bright field transmission microscopy picture was taken as a reference.

### Callose synthase assay

Incorporation of UDP-glucose into ammonium acetate- and ethanol-insoluble products was performed in Assay medium 2 (100 mM HEPES/KOH, 100 mM mannitol, 50 mM KCl, 0.5 mM EGTA, and 2 mM dithiothreitol (DTT), pH 7.5). Unless otherwise denoted, CaCl_2 _was added to 1 mM. In experiments investigating the cation requirements, CaCl_2 _and MgCl_2 _was added to Assay medium 2 as described in Fig. 4A. Cells washed and diluted to 40 mg (FW) ml^-1 ^in Assay medium 2 were incubated with alamethicin or digitonin (Fluka, recrystallized, Buchs, Switzerland) for 10 min. During incubation and the subsequent assay, samples were kept at room temperature on a rotary shaker (100 rpm). The reaction was started by addition of UDP- [^3^H]-glucose (18.5 GBq mol^-1^) to a final concentration of 0.5 mM, and was stopped by boiling after 10 min. Reactions where substrate was added after boiling was used as controls. Samples were transferred to 3 MM Whatman filter papers and washed with 4 ml per filter of a buffer containing 0.5 M ammonium acetate (pH 3.6) and 30% ethanol (v/v) using a sampling manifold (Millipore, Billerica, MA, USA). After drying for 30 min at room temperature, analysis of radioactively labeled product was performed as described [71] Pretreatment with 100 nM isoxaben (Riedel-de Haën, Seelze, Germany) was performed in Assay medium 2 for 10 min before alamethicin incubation. In experiments where cells had been pretreated with cytochalasin, DMSO, isoxaben, taxol or oryzalin in the growth medium, these chemicals were also present during the assay. In experiments where the effect of ethanol on UDP-glucose incorporation was investigated, the ethanol was included in the medium during the assay (final concentration 0.06% [v/v]). Where the alamethicin concentration was varied, solvent ethanol was kept constant at 0.012% (v/v).

### Aniline blue staining

BY-2 cells were washed once in Assay medium 2 and diluted to 40 mg (FW) ml^-1^. Cells were incubated with 20 μg ml^-1 ^of alamethicin for 10 minutes, after which EGTA was added to controls to a final concentration of 5 mM. The callose synthase assay was started by addition of UDP-glucose to 2 mM. After 10 min incubation at room temperature, the reaction was stopped by addition of EGTA to 5 mM to the non-control samples. Aniline blue and ethanol were added to the reactions to final concentrations of 0.05% and 50% respectively. After 30 min incubation, the staining solution was removed by centrifugation and the resulting pellet of cells was washed once in Assay medium 2 and mounted on glass slides. Stained cells were studied under a fluorescence microscope Nikon-Optiphot-2 microscope (Nikon Corporation, Tokyo, Japan) using a Nikon UV-1A filter (excitation at 360–370 nm, emission above 420 nm).

### Spectrophotometric enzyme activity determination

Cells were diluted to a density of 40 mg (FW) ml^-1 ^in Assay medium 1 before use and kept on stirring during the assay. Cells were incubated with alamethicin (20 μg ml^-1^) for 10 min after which 1 mM KCN and 50 nM n-propyl gallate was added (final concentrations). Enzyme activities were measured as absorbance changes of NAD(P)^+^/NAD(P)H at 340–400 nm in an Aminco DW-2a spectrophotometer using a stirred cuvette. All assays were started by addition of substrate.

PEPC and phosphorylating GAPDH, markers for cytosol, were assayed according to [72], and NAD-IDH, marker for mitochondria, was assayed according to [73]. For all three activities, the reaction mixture was supplemented with 100 mM KCl, 50 mM sucrose, 1 mM KCN, 50 μM n-PG and 1 mM EGTA. For NAD-IDH, the MgSO_4 _concentration was doubled to 2 mM. All reactions were started by the addition of the metabolite substrate When measuring G6PDH activities, NADP^+ ^(1 mM) and DTT (5 mM when included) were added before the assay was started by addition of glucose-6-phosphate to 2 mM final concentration. Cytosolic and plastidic activities of G6PDH were distinguished by that the plastidic, but not the cytosolic enzyme is inhibited by DTT [74].

### Immunofluorescence

Cultured cells were fixed and immunolabeled [75] with the modification that non-acetylated bovine serum albumin was used as blocking agent. For experiments analyzing alamethicin permeabilization, cells were washed once in Assay medium 2 and diluted in the same medium to 40 mg (FW) ml^-1 ^before alamethicin addition. Fixed cells were mounted on polylysine-coated microscope slides and the primary anti-β-tubulin antibody (N 357, Amersham BioSciences, Piscataway, NJ, USA) was used at a dilution of 1:200. As secondary antibody Alexa Fluor 488 goat anti-mouse antibody (Molecular Probes Inc. Carlsbad, CA, USA) was used at a dilution of 1:100. Microscopic analysis of slides was performed using an Imager Z1 fluorescence microscope (Zeiss, Stockholm, Sweden). Deconvolution was performed on image stacks using the Volocity software (Improvision, Coventry, England) using a calculated point spread function.

### Plasma membrane purification

Cells were suspended in extraction buffer (50 mM MOPS/KOH, pH 7.5, 5 mM EDTA, 330 mM sucrose, 5 mM ascorbic acid, 3 mM DTT, 1 mM phenylmethylsulphonyl fluoride, 0.6% (w/v) polyvinyl polypyrrolidone) and homogenized as described previously [64]. Crude extracts were filtered through a 150 μm net and centrifuged at 7,200 × g for 15 min at 4°C to remove cell debris, cell walls and nuclei. The supernatants were centrifuged at 40,000 × g for 1 h at 4°C to pellet the microsomal fraction. Plasma membranes were purified from microsomal fraction by partitioning in an aqueous polymer two-phase system [27]. A phase system of the following composition was used: 6.0% (w/w) dextran T 500, 6.0% (w/w) polyethylene glycol 4000, 330 mM sucrose, 5 mM potassium phosphate (pH 7.8) and 2 mM KCl. After partitioning, plasma membranes were washed in wash medium (10 mM HEPES/KOH, pH 7.5, 250 mM mannitol) and pelleted by centrifugation at 100,000 × g for 1 hour at 4°C.

### Membrane solubilization and two dimensional blue native SDS-PAGE

Plasma membrane proteins (100 μg/lane) were solubilized with 1% n-octyl-β-D-glucopyranoside (OG) in the presence of 750 mM amino caproic acid and 100 mM BisTris, pH 7.0. Solubilization was carried out at 4°C for 30 min under continuous mixing. Solubilized proteins were separated from the detergent-insoluble fraction by centrifugation at 100,000 × g for 1 hour at 4°C. To the supernatant, Coomassie G-250 (Serva Blau G-250, Serva Biochemica, Heidelberg, Germany) was added to a final concentration 0.8% (w/v).

Samples were loaded onto 4–14.5% gradient blue native PAGE and subjected to native electrophoresis at 4°C [76]. Molecular mass markers were from Amersham Pharmacia (Amersham BioSciences, Uppsala, Sweden). When the native electrophoresis was finished, the lanes were cut out and denatured in 1% (w/v) SDS, 1% (v/v) β-mercaptoethanol for 5 min. After rinsing the lanes with distilled water they were mounted between glass plates and separation in the second dimension was performed in a 10% TrisTricine SDS-gel with a 6% stacking gel (Jänsch et al. 1996). Molecular mass markers were from BioRad (BioRad Laboratories, Hercules, CA, USA).

### Immunoblotting

Proteins separated on the second dimension gels were transferred onto a polyvinylidene difluoride membrane (Millipore) by wet electroblotting (Bio-Rad Laboratories, Hercules, CA USA). The blots were probed with the following primary antibodies: monoclonal anti-actin (ICN, USA) diluted 1:1,000, monoclonal anti-β-tubulin (Amersham BioSciences) diluted 1:1,000, polyclonal anti maize sucrose synthase (SS2) [77] diluted 1:500, and polyclonal anti callose synthase from *Nicotiana alata *[78] diluted 1:500. Immunodetection was performed using the enhanced chemiluminescence assay with secondary antibodies according to the Amersham ECL Western blotting protocol (GE Healthcare, Freiburg, Germany).

### Statistics

All values presented represent a minimum of two measurements each obtained from two separate biological replicates. To test difference between treatments, Student's t-test was used when comparing normalized values while pair-wise Students t-tests was used for non-normalized values. Where applicable: * = p < 0.05, ** = p < 0.01, *** = p < 0.005. Excel was used for statistical calculation.

## Abbreviations

SDS-PAGE: Sodium dodecyl sulfate-polyacrylamide gel electrophoresis; BY-2: tobacco Bright Yellow-2; Col-0: Arabidopsis thaliana Columbia-0; DMSO: dimethyl sulfoxide; DTT: dithiothreitol; ER: endoplasmic reticulum; G6PDH: glucose-6-phosphate dehydrogenase; GAPDH: glyceraldehyde-3-phosphate dehydrogenase; FW: fresh weight; NAD-IDH: NAD-isocitrate dehydrogenase; PEPC: phosphoenolpyruvate carboxylase.

## Authors' contributions

SW and MA conceived the study. Experiments were planned and results interpreted mainly by MA, SW and AGR. MA conducted all experiments except for Fig. 1 that was conducted by CJA. SW and MA wrote the manuscript with substantial contribution also from AGR. All authors read, commented and approved the manuscript.
